# Harnessing theta waves: tACS as a breakthrough in alleviating post-stroke chronic pain

**DOI:** 10.3389/fnins.2025.1553862

**Published:** 2025-04-30

**Authors:** Ningjing Song, Ling Long, Nianquan Liu, Yujun Luo, Meng Wei, Hai Huang, Wan Liu

**Affiliations:** ^1^Department of Tuina and Rehabilitation Medicine, Hubei Provincial Hospital of Traditional Chinese Medicine, Wuhan, China; ^2^Department of Tuina and Rehabilitation Medicine, Affiliated Hospital of Hubei University of Chinese Medicine, Wuhan, China; ^3^Hubei Sizhen Laboratory, Wuhan, China; ^4^First Clinical Medical College, Hubei University of Chinese Medicine, Wuhan, China

**Keywords:** post-stroke chronic pain, sensorimotor cortex, tACS, theta waves, neural oscillations, mechanism

## Abstract

Neural oscillations play a critical role in the regulation of brain functions, with theta waves (4–8 Hz) in the sensorimotor cortex significantly influencing pain perception and modulation. These oscillations can modulate pain signal transmission, emotional cognition, and neuroplasticity. Post-stroke chronic pain is a common and complex symptom that imposes significant physiological and psychological burdens on patients. Transcranial alternating current stimulation (tACS), a non-invasive brain stimulation technique, can synchronize specific frequency neural activities, reorganize brain networks, and modulate neuroplasticity by adjusting specific frequency neural oscillations. In recent years, tACS has been widely applied in the research and treatment of various neurological and psychiatric disorders. This study aims to systematically summarize the current research progress on the regulation of θ oscillations in sensorimotor cortex by tACS. By reviewing relevant experimental and clinical studies, we explore the specific mechanisms of θ oscillations in pain perception and modulation and analyze the mechanisms and effects of tACS modulation of θ oscillations. Additionally, we examine the central and peripheral neural mechanisms of post-stroke chronic pain, emphasizing the critical role of the sensorimotor cortex in pain processing. In conclusion, tACS shows potential for modulating sensorimotor cortex θ oscillations and alleviating post-stroke chronic pain. This research provides new insights into the neural modulation mechanisms related to pain and offers potential new directions for developing novel therapies. Future clinical studies and technological optimizations are necessary to ensure the effectiveness and feasibility of tACS in clinical practice.

## 1 Introduction

Chronic post-stroke pain (PSCP) is a prevalent complication, affecting approximately 12% of individuals who have experienced a stroke (Zhan et al., 2019). The pathogenesis of PSCP is intricate, involving the reorganization and dysfunction of both the central and peripheral nervous systems (Larson et al., 2019). Patients frequently endure severe neuropathic pain, sensory abnormalities, and heightened pain sensitivity. Current therapeutic interventions, including pharmacological treatments, physiotherapy, and cognitive behavioral therapy, often demonstrate limited efficacy, with many patients struggling to achieve sustained pain relief (Haslam et al., 2021). Central nervous system pathological alterations are central to the challenging nature of PSCP, particularly the dysfunction of the sensorimotor cortex, which is closely linked to pain perception. Consequently, the modulation of sensorimotor cortex activity to alleviate pain has emerged as an urgent research priority. Recent advancements in neuroscience have highlighted the significant role of neural electrical activity in pain stimulation and perception (Yin and Zhao, 2024). Consequently, the modulation of sensorimotor cortex activity to alleviate pain has emerged as an urgent research priority.

Recent advancements in neuroscience have highlighted the significant role of neural electrical activity in pain stimulation and perception, emphasizing the importance of neural oscillation energy regulation and phase properties in the onset and modulation of pain. Neural oscillations are integral not only to pain processing but also to a wide range of cognitive and sensory functions. The sensorimotor cortex, a critical region for processing sensory input and motor output (Kong et al., 2024), is particularly influenced by θ-wave (4–8 Hz) neural oscillations (θ oscillations), which are pivotal in sensorimotor integration, attention regulation, and pain modulation. Research indicates that synchronized θ wave activity may enhance the integration of sensory information and the formulation of motor plans by modulating the functional connectivity within the sensorimotor network. Furthermore, θ waves play a distinctive role in pain modulation, particularly in chronic pain conditions, where abnormalities in θ wave patterns may be linked to central sensitization phenomena. Such abnormalities in neural oscillation patterns are posited to be central mechanisms in the development of chronic pain.

Transcranial alternating current stimulation (tACS) is an emerging neuromodulation technique that non-invasively applies specific frequency alternating currents to the scalp to modulate neural oscillations in the brain (Feurra et al., 2011; Wischnewski et al., 2023). Transcranial alternating current stimulation (tACS) presents a promising avenue for the treatment of persistent sensorimotor cortex pain (PSCP) through the modulation of θ oscillations. Although the application of tACS in pain research remains in its nascent stages, ongoing investigations continue to explore its analgesic effects and underlying mechanisms, with a current paucity of direct evidence to definitively elucidate the analgesic mechanism of tACS (Angelakis et al., 2013; May et al., 2021). This study aims to systematically synthesize the existing research on the modulation of θ oscillations in the sensorimotor cortex via tACS, to investigate the specific role of these oscillations in pain perception and modulation, and to analyze the effects and mechanisms of their modulation by tACS. Furthermore, this paper will delve into the mechanisms of tACS intervention in PSCP, with a particular focus on the central and peripheral neural mechanisms involved, highlighting the critical role of the sensorimotor cortex in pain processing. Additionally, the paper will propose future research directions and discuss the clinical application prospects of tACS, with the objective of providing a theoretical foundation and practical references for the treatment of chronic pain using this modality.

## 2 Neural oscillations

### 2.1 Definition and classification of neural oscillations

Neural oscillations are periodic fluctuations generated by groups of neurons in the brain, through electrical activity. These synchronized oscillations reflect the synergistic interactions among neurons and serve as a foundation for information processing within the brain (Wang et al., 2020). Research has demonstrated that neural oscillatory mechanisms are pivotal to understanding neural networks in the context of chronic pain. The frequency, amplitude, and phase of these oscillations represent local, network, and even whole-brain states that not only influence immediate neuronal responses but may also induce changes in synaptic plasticity, affecting neuromodulatory outcomes (Sadaghiani et al., 2010). Fu et al. (2018) observed that acute mechanical pain stimulation during chronic pain conditions resulted in an increase in α oscillations, a decrease in β, γ, and δ oscillations, and a dynamic reduction in γ oscillations. Furthermore, it has been shown that injurious stimuli lead to a decrease in the power of α and β oscillations, an increase in γ oscillations (Ploner et al., 2006; Hauck et al., 2015) and an enhancement in θ-γ coherence (Wang et al., 2011). Neural oscillations across various frequency bands, along with their synchronization both within specific frequencies and across distinct brain regions, facilitate complex cognitive processes (Neske, 2015). These oscillatory patterns not only represent brain activity across different states but are also intricately linked to a wide range of functions, including perceptual, motor, and cognitive tasks. The phase of sustained oscillations is indicative of cortical processing of threshold visual stimuli, thereby establishing a direct connection between oscillatory phases and sensory perceptions and behaviors. The brain orchestrates the activities of diverse regions by modulating neural oscillations at varying frequencies. Neural oscillations are typically categorized into frequency bands based on their frequency range, with each band associated with specific brain functions (Table 1). The amplitude energy of high-frequency rhythms is closely associated with alterations in synaptic activity, while phase changes in low-frequency rhythms reveal the excitatory state of individual neurons or neuron populations. Each frequency band of neural oscillations contributes to distinct cognitive and sensorimotor functions, the synergy between these elements forms the foundation for the realization of complex brain functions (Xiao et al., 2019; Chen et al., 2022).

**Table 1:** Definition and classification of neural oscillations.

Typology	Frequency range	Function	Brain area involved
δ	1–4 Hz	Deep sleep, memory integration, restorative brain functioning	Brainstem, thalamus, cerebral cortex
θ	4–8 Hz	Memory, emotion regulation, motor control, pain regulation	Sensorimotor cortex, hippocampus, prefrontal cortex
α	8–12 Hz	Cognitive processing, attention allocation, and introverted thinking in the resting state	Occipital cortex, parietal cortex
β	13–30 Hz	Motor preparation, perceived motor continuity, cognitive processing	Motor cortex, frontal cortex, basal ganglia
γ	30–100 Hz	Higher-order cognitive functions, perceptual integration, rapid information processing, consciousness	Cortico-cortical connections, mainly in sensory and visual areas

### 2.2 The role of θ oscillations in brain function

**Figure 1:**
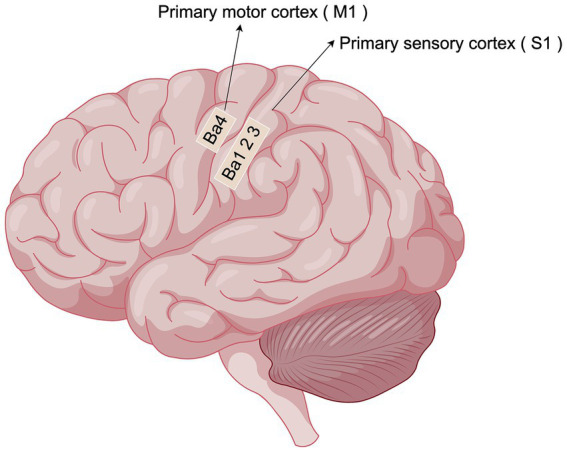
Localization of sensorimotor cortex (By Figdraw). The sensory-motor cortex mainly consists of two important subdivisions, S1 and M1: S1 is located in the posterior central gyrus of the parietal lobe of the brain, behind the central sulcus. It is mainly responsible for receiving and processing touch, temperature, pain and proprioception (somatosensation) from the whole body.M1 is located in the precentral gyrus of the frontal lobe of the brain, in front of the central sulcus. Primarily controls random movements, sending motor commands to the spinal cord and muscles.

θ waves typically manifest in various brain regions during tasks such as memory processing, emotion regulation, and navigation. In the sensorimotor cortex, θ oscillations primarily function to modulate motor control, integrate sensory inputs, and process pain. These neural oscillations play a central role in the sensorimotor cortex by not only modulating pain perception but also optimizing motor and sensory functions. The sensorimotor cortex is crucial for integrating sensory inputs and controlling motor outputs (Figure 1). θ oscillations in this region are involved in the planning and execution of movements, as well as the integration of sensory feedback (Ponsel et al., 2020). Research indicates that θ waves are essential for coordinating the temporal precision of sensory information and motor output, serving a synchronizing role in the regulation of fine motor movements. θ waves are instrumental in facilitating effective communication pathways between various cortical regions during the execution of fine motor tasks, thereby enhancing coordination and feedback regulation of movement. Moreover, θ oscillations hold a distinctive role in pain perception and modulation. Patients suffering from chronic pain frequently display atypical θ wave activity (Stern et al., 2006), particularly in the context of central sensitization, where dysfunction of θ oscillations within pain networks may be linked to persistent pain perception (Sarnthein et al., 2006). This observation indicates that modulating θ oscillations in the sensorimotor cortex could serve as a potential strategy for pain alleviation. Furthermore, θ waves are crucial in multiple brain regions beyond the sensorimotor cortex (Table 2).

**Table 2:** The role of θ oscillations in brain function.

Brain region	Mechanism	Function
Sensorimotor Cortex	Sensory input synchronized with motor output	Motor control, sensory feedback, pain perception
Hippocampus	Coordination of neuronal activity, long range connectivity of brain areas	Spatial navigation, memory encoding, short-term to long-term memory conversion
Prefrontal cortex	Complex Cognitive Functional Connectivity	Working memory, decision-making processes, emotional control, attention
Limbic system	Emotional processing, stress response	Emotion regulation, control of anxiety, stress response
Cingulate gyrus	Self-perception, conflict monitoring	Emotion regulation, conflict monitoring, integrating emotional cognition
Parietal cortex	Sensory information integration	Sensory integration, attention allocation, multisensory processing

## 3 Transcranial alternating current stimulation (tACS)

### 3.1 tACS technical characteristics and application mechanisms

**Figure 2:**
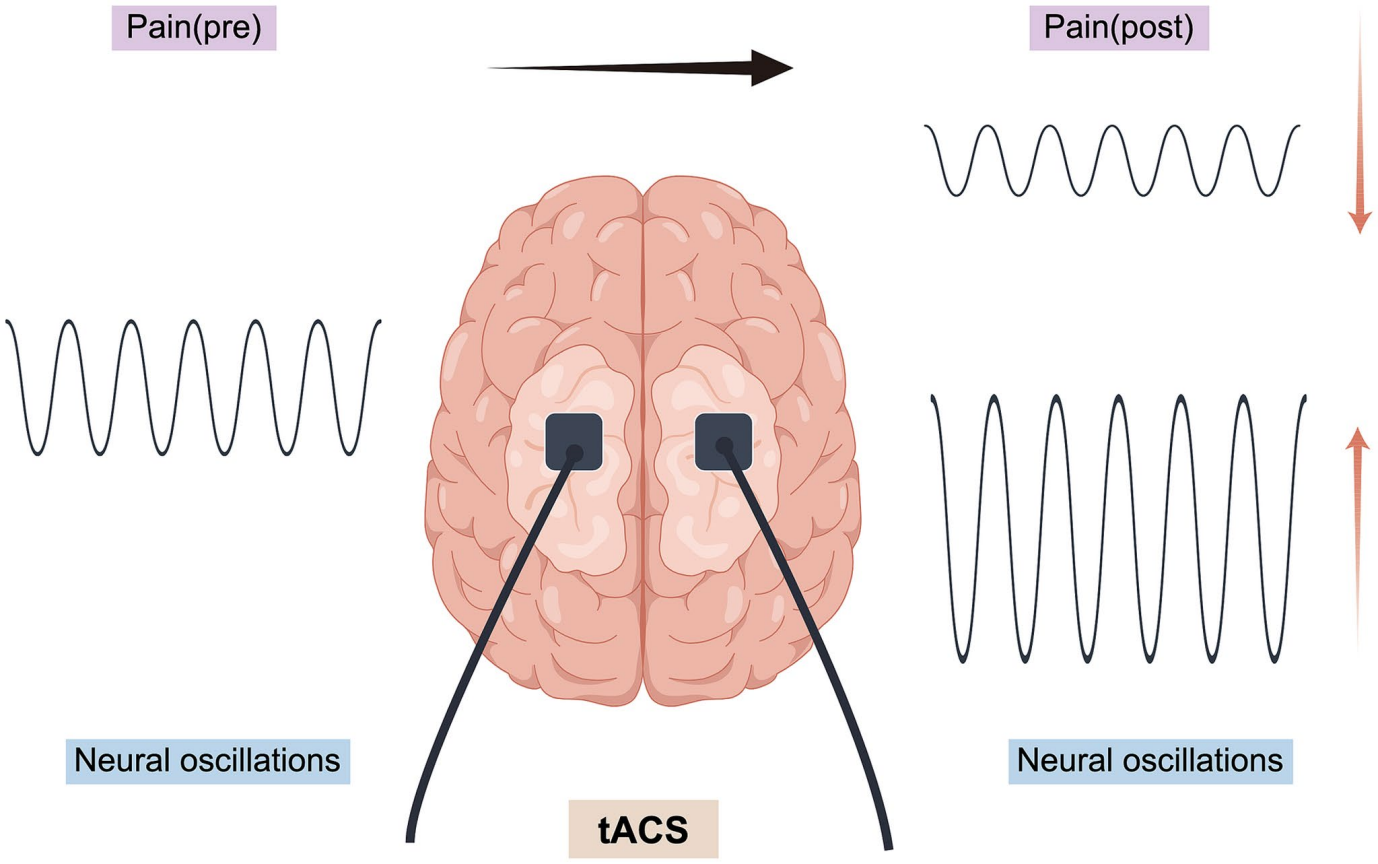
Application mechanism of tACS (By Figdraw). Upregulation: refers to the enhancement of neural oscillations at a specific frequency by tACS, whereby the application of an external AC stimulus matching the endogenous oscillations of a target brain region increases its amplitude or functional connectivity, synchronizes neural activity at that frequency, and thus potentially enhances the effect of that oscillatory pattern, which in turn enhances the strength of oscillations or functional connectivity in that brain region. Downregulation: refers to the inhibition of neural oscillations at a specific frequency by tACS by applying a current in the opposite phase of the target oscillation (phase interference), which reduces its amplitude or functional connectivity, thus potentially decreasing the contribution of that oscillatory pattern to pain.

tACS is a non-invasive brain stimulation (NIBS) technique characterized by non-invasiveness, high safety, frequency-specific modulation, and neural entrainment effects. tACS is able to enhance, inhibit, or remodel brain-specific frequency neural oscillations through the interaction of applied alternating currents with endogenous neural oscillations, and thus modulate neuronal activity (Guleyupoglu et al., 2013; Polania et al., 2018). tACS can use different waveforms to optimize the modulation effect on neural activity, and common waveforms include Sine Wave, Square Wave, Triangle Wave, Pulse Wave, Pseudo-random Wave, etc. (Table 3) (Bikson et al., 2019; Hsu et al., 2021; Sahu et al., 2021). The stimulation effect of tACS depends on whether the phase of the stimulation current matches the endogenous neural activity, i.e., Phase-dependent Modulation (PDM), when tACS is synchronized with endogenous oscillations, it can enhance neural activity, and when it is in anti-phase with neural oscillations, it can inhibit neural activity. Modulation (Phase-dependent Modulation). tACS can enhance neural activity when synchronized with endogenous oscillations, and inhibit neural activity when in anti-phase with neural oscillations (Tavakoli and Yun, 2017).The phase of sustained neural oscillations serves as an indicator of the cortical threshold response in the processing of visual stimuli, thereby elucidating a direct relationship between oscillatory phase and sensory perception and behavior. The impact of transcranial alternating current stimulation (tACS) on neuronal membrane potentials is subject to fluctuation due to the continuous alteration in the direction of the applied current (Vosskuhl et al., 2018). Throughout this process, neuronal membranes experience rapid transitions between hyperpolarization and depolarization, which may modulate the temporal pattern and frequency of neuronal firing, ultimately facilitating the synchronization of neural activity (Zaehle et al., 2010; Joundi et al., 2012; Antal and Herrmann, 2016) (Figure 2). In addition, tACS is able to modulate synaptic plasticity by altering the electrical excitability of neurons, affecting both long time-range potentiation (LTP) and long time-range depression (LTD) (Vogeti et al., 2022). It is noteworthy that the electric field generated by tACS does not directly trigger neuronal action potentials, but rather makes neurons easier or harder to be naturally activated by modulating the depolarized or hyperpolarized state of membrane potentials. This effect acts for a long time at the subthreshold level (subthreshold level) and may promote long-term plasticity changes (Korai et al., 2021).

**Table 3:** Classification of tACS waveforms.

Waveforms	Function	Application
Sine wave	Nerve entrainment, enhancement of endogenous oscillations	Cognitive enhancement, attention regulation, emotional regulation, sports rehabilitation
Square wave	Enhance neuroplasticity	Motor recovery, sensory modulation, synaptic plasticity enhancement
Triangular wave	Modulation of slow wave oscillations	Sleep regulation, chronic pain, motor recovery
Pulse wave	Precise regulation of neural networks	Epilepsy, Parkinson’s disease, neural synchronization
Pseudo-random wave	Mimic natural neural activity, reduce side effects	Parkinson’s disease, chronic pain, enhance neuroplasticity
Dual-frequency coupled wave	Influence cross-frequency brain connectivity	Memory enhancement, schizophrenia, Alzheimer’s disease
Stepped wave	Gradual modulation of neural activity	Long-term neuromodulation, reduction of skin irritation

Neural entrainment effect is one of the mechanisms of tACS regulation. Neural entrainment effect refers to the phenomenon in which external periodic stimuli (e.g., electrical stimulation, sound, light flicker, etc.) interact with endogenous oscillations of neurons to gradually synchronize neuronal activity with the frequency of external stimuli. This effect is widespread in the information processing process of the brain and has an important role in tACS and perceptual cognition research (Vogeti et al., 2022; Manippa et al., 2024). It is mainly manifested in three aspects: Frequency Matching, Phase Locking and Neuroplasticity: when an external stimulus (e.g., sinusoidal current in tACS) is applied at a specific frequency, neural oscillations in the brain may gradually adjust their own activity patterns and synchronize with the external stimulus. Synchronization. The researchers’ study of non-human primates confirmed tACS’ success in entraining neuronal activity (Krause et al., 2019; Vieira et al., 2020). In turn, the firing pattern of neurons may be synchronized with the phase of the external stimulus, thus affecting the temporal organization of neural activity. Prolonged entrainment effects may lead to changes in synaptic plasticity and functional readjustment of neural circuits (Luckey et al., 2022). Unlike other types of NIBS, the advantage of tACS lies in its ability to manipulate and entrain intrinsic oscillations by physiological induction through frequency stimulation with barely perceptible current intensity to enhance or inhibit the functioning of specific brain regions, such as alleviating neurological disease symptoms such as pain, numbness, etc., or boosting memory and cognition, which can enhance neuroplasticity (Thut et al., 2011; Tavakoli and Yun, 2017; Lakatos et al., 2019).

tACS directly influences neuronal activity, and alterations in neural oscillations within the brain can be observed several minutes or even up to 1 h following the conclusion of the intervention, a phenomenon referred to as the ‘offline effect’, is hypothesized to be associated with transient alterations in neuroplasticity (Zaehle et al., 2010; Kasten and Herrmann, 2017). While the majority of transcranial alternating current stimulation (tACS) research has traditionally concentrated on modulating ongoing rhythmic brain activity at specific frequencies, recent investigations have revealed that tACS is not confined to the modulation of a single frequency. Instead, it can also influence the interaction between different frequencies. These inter-frequency interactions may be crucial in various neural processes and cognitive functions, thereby highlighting the potential of tACS in neuromodulation. In addition, tACS not only acts on individual neurons, but also affects functional connectivity between different regions of the brain. For example, transregional bipolar tACS can synchronize neural oscillations in two brain regions, enhancing their interactions and facilitating information transfer. By precisely controlling phase coupling between distinct frequency bands, tACS holds the capability to affect the synergistic activity across different brain regions (Herrmann et al., 2016). In recent years, researchers have effectively addressed the conventional limitations of tACS, particularly the challenge of targeting specific brain regions. This has been accomplished through innovative methodologies, including high-definition tACS, phase-shifted tACS, amplitude-modulated tACS, time-interference (TI) techniques, and intersecting short pulses (ISPs) (Huang and Parra, 2019; Saturnino et al., 2019; Wu et al., 2021). Notably, long-term tACS stimulation may even induce structural neuroplasticity changes, such as promoting synaptic growth and enhancing the stability of neuronal networks. Recent advancements have propelled tACS to a more sophisticated stage of development, enhancing its potential for the treatment and alleviation of pain symptoms.

In summary, tACS affects the nervous system through a variety of mechanisms, including interaction with endogenous neural oscillations, modulation of synaptic plasticity, remodeling of functional connectivity, alteration of membrane potential, modulation of neurotransmitters, and neural entrainment effects. Together, these mechanisms make tACS a promising tool for neuromodulation, which is widely used in the fields of neurorehabilitation, cognitive enhancement, and treatment of psychiatric disorders.

### 3.2 Role of tACS in pain modulation

Pain is a highly subjective experience, influenced not only by external stimuli but also by an individual’s cognitive, emotional, and motivational background factors, which are dynamically integrated in the brain to form a complex pain perception process. Although the experience of pain is universal, there is no specific ‘pain center’ in the brain dedicated to processing this phenomenon. Instead, pain processing involves the synergistic interaction of multiple functional areas, each originally responsible for different tasks. The transmission of pain signals among these brain regions is dynamic, occurring across various time scales and frequencies, illustrating how the brain adapts pain perception and coping mechanisms to the diverse physiological and psychological states of individuals (Ploner et al., 2017; Cecchi, 2020). tACS can precisely modulate neural activity across different frequency bands in the brain, thereby influencing the synchronization of neural networks and the process of information transfer. The application of alternating current (AC) with a fixed frequency and amplitude to the brain can modulate the neural oscillation patterns, thereby altering the brain’s pain response mechanisms to some degree. This approach offers a novel non-pharmacological therapeutic pathway for pain management (Ahn et al., 2019; Hohn et al., 2019; Meeker et al., 2020; Alfihed et al., 2024). It can be utilized to explore the relationship between observed oscillatory brain activity and various pain types and dimensions by modulating endogenous pain-related oscillatory brain activity (either up-regulation or down-regulation) and assessing whether this modulation results in changes in pain perception (see Figure 2). Although transcranial alternating current stimulation (tACS) and transcranial direct current stimulation (tDCS) employ similar equipment, they differ fundamentally in their methods of electrical stimulation. tACS is distinguished by its use of AC current aimed at modulating neural oscillation patterns at a specific frequency, whereas tDCS influences neuronal membrane potentials through the application of a fixed-intensity direct current (DC). In contrast to transcranial random noise stimulation (tRNS), transcranial alternating current stimulation (tACS) employs a constant stimulation frequency, facilitating precise modulation of neural oscillations across various brain frequencies. This distinct characteristic renders tACS particularly advantageous for neuromodulation, enabling more accurate regulation of neural activity and enhancing cognitive, emotional, and perceptual functions (O'Connell et al., 2018; Yao et al., 2021; van der Groen et al., 2022). Ahn et al. (2019) demonstrated a significant correlation between the enhancement of alpha oscillatory signaling via tACS and its analgesic effects, thus supporting the hypothesis that tACS modulates pain perception through the alteration of neural oscillatory signaling. Although electrical stimulation has been shown to improve motor function and alleviate chronic pain post-injury, it is limited by low spatial resolution and difficulties in selectively targeting individual neurons. Consequently, further research is necessary to elucidate the pain-modulating effects and underlying mechanisms of tACS.

## 4 Neural mechanisms of PSCP

PSCP is a complex and challenging pathology to manage, clinically categorized into central post-stroke pain (CPSP), hemiplegic shoulder pain, spastic pain, complex regional pain syndrome, and headache. The neural mechanisms underlying PSCP involve intricate interactions between the central and peripheral nervous systems. The development of PSCP is closely associated with central neural network dysfunction, abnormal neural oscillations, and changes in peripheral nerve signaling.

### 4.1 Central mechanism

CPSP, a disabling and incurable form of central neuropathic pain, affects approximately 8 to 55% of stroke patients. The pooled prevalence of CPSP in patients with stroke at any location was 11%, and CPSP manifests within a month since symptom onset in 31% of patients (Liampas et al., 2020). Characterized by persistent or intermittent neuropathic pain. This condition results from complex and dynamic dysfunctions across multiple brain regions, involving both localized neural activity abnormalities and disrupted functional connectivity within brain networks (Roosink et al., 2010; Choi et al., 2021). These central abnormalities collectively contribute to the dysregulated processing and amplification of pain signals, as well as adverse emotional and cognitive experiences, culminating in a chronic pain condition that is challenging to alleviate. This condition is frequently associated with atypical temperature and/or pressure sensations, alongside anxiety and depression (Corbetta et al., 2018; Shi et al., 2023). The mechanisms underlying central pain are hypothesized to include spinal thalamic dysfunction, medial thalamic disinhibition, neuronal hyperexcitability in thalamocortical regions, and afferent neurotomy. Pain processing is mediated by a complex central neural network encompassing the sensorimotor cortex (S1/M1), anterior cingulate cortex (ACC), limbic system, thalamus, and prefrontal cortex (PFC). These brain regions are integral to pain perception and pain-related neural processing through the establishment of functional connections that constitute a pain network (Li et al., 2017). Recent studies employing diffusion tensor imaging have identified structural alterations in the white matter of pain-processing regions, such as the thalamus, somatosensory cortex, and cingulate and insula cortex regions (Gritsch et al., 2016). The sensorimotor cortex is a fundamental region involved in the perception and modulation of pain. Its functions encompass receiving nociceptive signals from peripheral inputs, assessing pain intensity, and engaging in pain modulation. The equilibrium between excitatory and inhibitory neurotransmitters is crucial in the conduction of pain signals by nerve fibers within the somatosensory system, influencing both the efficiency and intensity of pain signal transmission. The mechanism of action of the sensorimotor cortex in chronic pain after stroke involves a combination of neural reorganization, amplification of pain signals, imbalance of pain modulation, reorganization of the sensory cortex, and neuroinflammatory and neuropathic pain (Kanika and Goyal, 2023). In patients with stroke, the sensory-motor cortex may be functionally abnormal or reorganized as a result of local ischemia or neuronal injury, which may lead to disturbances in the processing of pain signals (Bushnell et al., 2013; Argoff, 2024). In addition, due to the stroke increase in excitatory neurotransmitters (e.g., glutamate) and decrease in inhibitory neurotransmitters (e.g., GABA) after stroke, which leads to an increase in the efficiency and intensity of pain signaling and the appearance of neuroinflammation and neuropathic pain, the sensory-motor cortex may amplify the pain signals, resulting in more intense pain felt by the patient, which further exacerbates the perception and persistence of pain (Kuner and Flor, 2017; Lekoubou et al., 2023). Presently, research on the mechanisms underlying central post-stroke pain (CPSP) is limited. Although advancements in functional brain imaging and the identification of factors predisposing individuals to central pain have enhanced the understanding of several aspects contributing to the onset of CPSP, comprehensive knowledge of the pathophysiology of neuropathic pain following stroke remains inadequate, rendering its treatment challenging.

Central sensitization is one of the core mechanisms of chronic pain and involves an abnormal response of the central nervous system (CNS) to pain signals, which manifests itself as increased excitability of neurons in the spinal cord and cerebral cortex, leading to amplification of pain signals and decreased nociceptive modulation (Ji et al., 2018). Particularly in patients with post-stroke chronic pain (PSCP), the process of central sensitization is often accompanied by dysfunction of CNS networks and the appearance of abnormal neural oscillatory activity. Imbalances in these neural networks are closely associated with the persistence and exacerbation of pain. In the context of chronic pain after stroke, central sensitization is not only due to abnormal transmission of peripheral nociceptive signals, but is also closely related to changes in neural oscillations and functional connectivity within the CNS (van Griensven et al., 2020; Volcheck et al., 2023). In patients with PSCP, the sensorimotor cortex frequently demonstrates abnormal low-frequency neural oscillations, such as theta and delta wave activities. These oscillations are intricately linked to the amplification of pain signals and the impaired regulation of these signals. Such low-frequency oscillations are considered a maladaptive neural response to prolonged pain exposure and are often associated with the chronicity of pain, reduced pain tolerance, and heightened mood disorders. The phenomena of central sensitization extend beyond the sensorimotor cortex, implicating other critical regions of the central nervous system, notably the thalamus, anterior cingulate gyrus (ACC), and prefrontal cortex. The thalamus, in particular, plays a pivotal role in patients with PSCP, it serves as a crucial relay station for the transmission of nociceptive information, acting as a pivotal node for the conveyance of pain signals from the periphery to the central nervous system (Treister et al., 2017). Abnormalities within central pain pathways are evident as dysfunctions in the spinal thalamic tracts, which may cause damage to localized thalamic structures or lead to aberrant neural pathways. These alterations can result in either an abnormally heightened transmission of nociceptive signals or a diminished inhibition of pain signals. Such abnormal transmission further intensifies the persistence and severity of pain, particularly within the spinal thalamic tracts, which are essential components of the sensory pathways responsible for somatic pain, thermal sensation, and tactile perception (Mohanan et al., 2023).

In contrast, the anterior cingulate gyrus plays a crucial role in the emotional and cognitive regulation of pain, with its primary functions encompassing the modulation of emotional responses to pain and the allocation of attention. Post-stroke, the activity within the anterior cingulate gyrus may be anomalously impacted, as indicated by hyperactivation or altered functional connectivity. This abnormality intensifies the patient’s adverse emotional experience of pain and may disrupt the mechanisms of emotion regulation, thereby amplifying the subjective perception of pain (Hauck et al., 2015). The limbic system, which includes regions such as the hippocampus and amygdala, is intricately linked to emotional responses to pain and memory formation (Buzsaki and Moser, 2013). Following a stroke, these areas may experience exacerbated abnormal functional connectivity with the sensorimotor cortex and prefrontal cortex, resulting in an increased experience of pain-related emotions and potentially inducing a chronic pain state. The prefrontal cortex is integral to the cognitive modulation of pain and the development of coping strategies, particularly in relation to attention allocation and pain tolerance (Huishi Zhang et al., 2016; May et al., 2019). Following a stroke, functional impairment of the prefrontal cortex may hinder patients’ ability to regulate their subjective perception of pain through cognitive resources, potentially exacerbating their pain experience.

By integrating the mechanisms of central sensitization with the components of abnormal neural oscillations and functional connectivity in chronic post-stroke pain, it becomes evident that these mechanisms are intricately interconnected. Central sensitization contributes to the persistence of pain by enhancing nociceptive signaling and diminishing pain modulation. Concurrently, abnormalities in neural oscillations reflect a maladaptive cerebral response to pain, which further exacerbates central sensitization. Moreover, imbalances in functional connectivity among brain regions, particularly the thalamus, anterior cingulate cortex, and prefrontal cortex, disrupt the emotional processing of pain and cognitive regulatory mechanisms. This disruption leads to an over-amplification of pain perception, perpetuating a vicious cycle.

Consequently, elucidating the relationship between central sensitization, neural network dysfunction, and aberrant neural oscillatory activity in the context of post-stroke chronic pain offers a multidimensional perspective for uncovering the physiological mechanisms underlying chronic pain following a stroke. This understanding also identifies a more precise target for future therapeutic interventions.

### 4.2 Peripheral mechanisms

Additionally, the peripheral nervous system plays a critical role in the generation and transmission of pain. Post-stroke, abnormal functioning of the peripheral nervous system may amplify pain signals through various mechanisms, thereby intensifying the persistence and chronicity of pain. Peripheral tissues may become sensitized due to local ischemia, inflammation, or metabolic abnormalities, leading to the sensitization of sensory nerve endings. This sensitization is characterized by heightened responses of peripheral nerve endings to mechanical, thermal, or chemical stimuli, resulting in the perception of even normal physiological stimuli as painful (i.e., low-threshold mechanical pain). Chronic pain is predominantly attributed to the release of pro-inflammatory cytokines, notably tumor necrosis factor-alpha (TNF-α), which is intricately linked with neuropathic pain. This process involves the release of inflammatory mediators that further activate or enhance the excitability of sensory nerves (Kuan et al., 2015; Liu et al., 2017). Neuronal hyperexcitability is induced by the release of the excitatory neurotransmitter glutamate at sites of nerve injury. Brain imaging studies have corroborated the presence of elevated glutamate concentrations under painful conditions (Hassaballa and Harvey, 2020). Conversely, substantial evidence indicates that the expression of the P2 receptor family isoform (P2 × 7) in microglia is upregulated in the thalamic ventral posterolateral nucleus (VPL) following hemorrhagic events. The P2 × 7 receptor, predominantly expressed in microglia, subsequently facilitates the specific release of interleukin-1β (IL-1β), interleukin-6 (IL-6), and TNF-α, along with other cytokines such as chemokine SDF-1. This cascade triggers the release of glutamate in regions adjacent to the stroke site. As a result, sensitized nerve endings persist in transmitting aberrant pain signals to the central nervous system, culminating in chronic pain (Di Virgilio, 2015; Huang et al., 2020; Li H. L. et al., 2023). This enhancement of peripheral-central transmission not only intensifies pain perception but may also precipitate widespread pain beyond the initial focal site.

Furthermore, hyperactivation of the sympathetic nervous system significantly contributes to the amplification of pain signals. Following a stroke, increased sympathetic activity may further potentiate the generation and transmission of peripheral pain signals through interactions with peripheral sensory nerve endings (Arslan and Unal Cevik, 2022). For instance, norepinephrine released by sympathetic nerves can directly activate receptors in sensory nerve endings, resulting in the excessive amplification of pain signals. The sustained activation of the sympathetic nervous system may establish a feedback loop with the central pain network, thereby intensifying and prolonging pain (Janig, 2014; Jewson et al., 2015). This interaction between peripheral and central systems can result in a self-perpetuating cycle that complicates pain alleviation. In stroke patients, peripheral nerve injury or degenerative changes can significantly contribute to the onset and maintenance of pain. For instance, abnormal nerve discharges or nerve sprouting following nerve injury may further amplify pain signals. Additionally, the regeneration of unmyelinated nerve fibers, often structurally incomplete after nerve injury, can lead to abnormal pain perception or persistent pain (Liu et al., 2023).

The peripheral nervous system plays a crucial role in post-stroke central pain (PSCP) through mechanisms such as the sensitization of sensory nerve endings, enhanced peripheral-central pain signaling, overactivation of the sympathetic nervous system, and peripheral nerve injury. Collectively, these mechanisms not only intensify pain but also influence its persistence and chronicity. Therefore, interventions aimed at the peripheral nervous system, such as inhibiting sensitization signals, modulating sympathetic nerve activity, or repairing peripheral nerve injuries, may offer effective therapeutic targets for the treatment of PSCP.

## 5 Mechanisms of tACS modulation of θ oscillations in the treatment of PSCP

### 5.1 Role of θ oscillations in pain modulation

**Figure 3:**
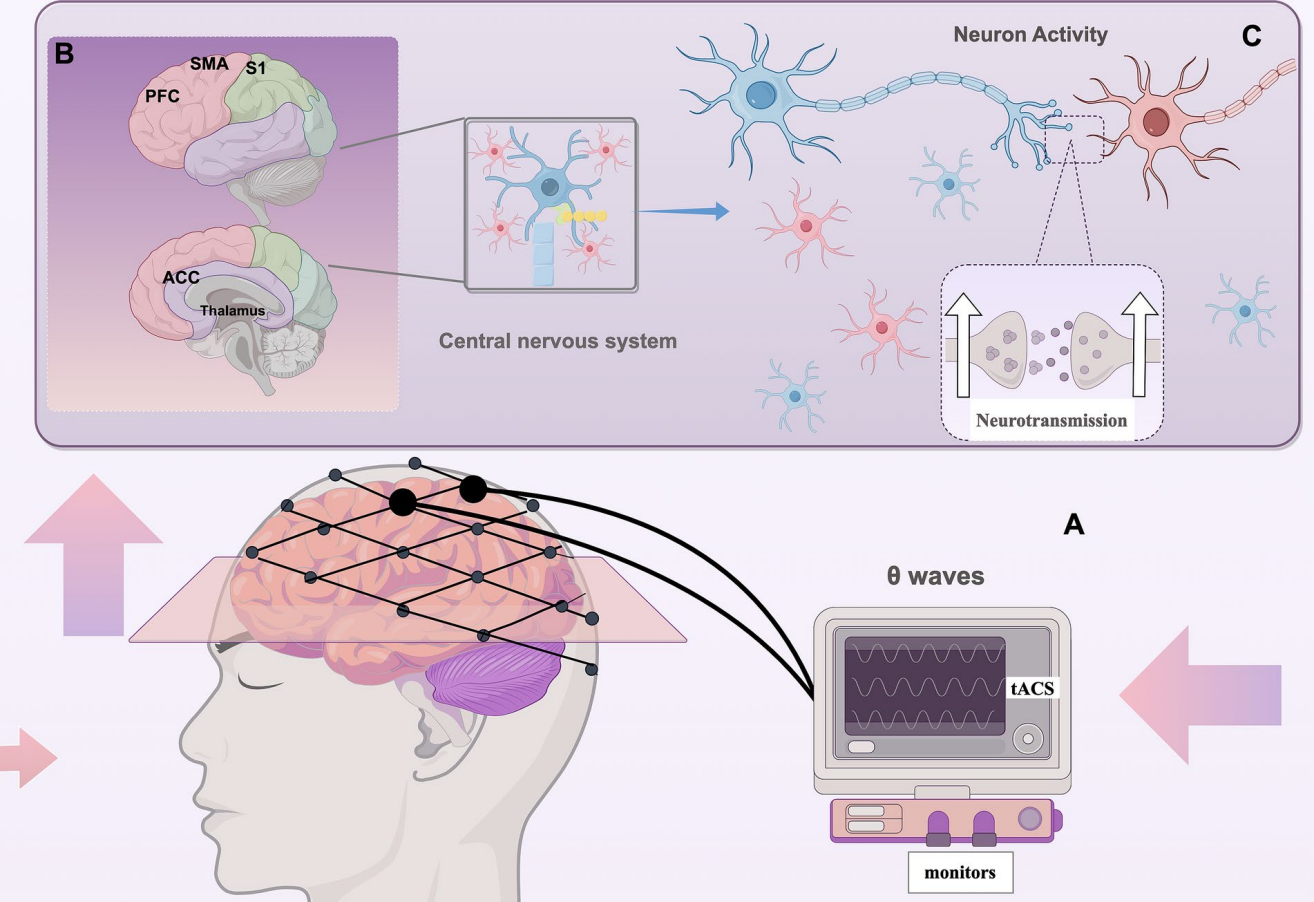
Regulation of pain by tACS (By Figdraw) **(A)** tACS applies sinusoidal AC current to the sensorimotor cortex to modulate θ oscillations; **(B)** Pain processing regions: S1/M1: Sensorimotor Cortex, ACC: Anterior Cingulate Cortex, PFC: Prefrontal Cortex, Thalamus, SMA: Supplementary Motor Area; **(C)** Enhancing theta wave function boosts brain connectivity, synchronizes neurons, improves neuroplasticity, and reduces abnormal pain signal amplification.

Studies have demonstrated that the functional connectivity between the brainstem and the anterior insular cortex influences susceptibility to pain (Ploner et al., 2010), θ waves are crucial in regulating the functional connectivity between the sensorimotor cortex and the thalamus. These oscillations may originate from brain regions overlapping with the somato-social pain matrix, including the somatosensory cortex, anterior cingulate cortex, frontal pole, and motor accessory areas. The perception and processing of pain signals rely on the coordinated activity among these regions. Sustained activity within the theta wave band is specific to pain and is linked to behavioral responses (Figure 3). Therefore, dynamic coordination across these regions via theta waves is essential for the joint response to and processing of pain signals (Taesler and Rose, 2016; Zhang et al., 2021). This activity may modulate the strength and efficiency of pain signaling by facilitating phase coupling between neurons (Watrous et al., 2013; Crespo-Garcia et al., 2016). Under typical physiological conditions, θ wave activity plays a crucial role in moderating the expression of pain signals and preventing excessive reactions. During the body’s processing of pain in response to injurious stimuli, fluctuations in the θ band may be induced (Schulz et al., 2011; Schulz et al., 2012). Post-stroke, the coordination within pain modulation networks may become disrupted, leading to abnormal θ wave activity or impaired connectivity in response to injurious stimuli. This disruption can result in the abnormal amplification and chronicity of pain signals, potentially triggering atypical pain perception (Haslam et al., 2023; Dawson et al., 2024). Furthermore, abnormal synchronization of θ waves may exacerbate patients’ negative emotional responses to pain. However, no study has yet conducted an in-depth investigation into the application of θ oscillations in post-stroke central pain (PSCP). Based on the current understanding of their application in pain intervention and the associated mechanisms, we hypothesize that following a stroke, as the functions of the sensorimotor cortex and other pain-related regions undergo reorganization, appropriate θ-wave activity could provide the necessary neural basis for such plasticity. Restoring θ wave function may enhance functional connectivity between the sensorimotor cortex and the thalamus, thereby mitigating the abnormal amplification of pain signals. Simultaneously, by promoting adaptive changes in neural networks, θ waves may facilitate the recovery of function in damaged regions, thereby alleviating chronic pain. Additionally, θ waves have been strongly associated with situational memory, cognitive control, and emotion regulation (Herweg et al., 2020; Gedankien et al., 2023). By modulating the activity of the prefrontal cortex and limbic system, they may assist patients in cognitively reappraising pain signals and emotionally alleviating them. Through a systematic review and meta-analysis of 56 studies, Lee et al. demonstrated that cognitive performance is enhanced in theta frequency bands within the prefrontal and posterior parietal cortical regions during both online and offline transcranial alternating current stimulation (tACS). Furthermore, both modalities of tACS at theta frequency bands were found to enhance executive function. These findings suggest that tACS, when applied with specific timing and frequency parameters, may be effective in improving cognitive performance (Lee et al., 2023). Following a stroke, the role of θ waves may be diminished, increasing the patient’s sensitivity to pain signals and exacerbating pain symptoms. Consequently, alleviating pain-related emotional distress can be achieved, to some extent, by restoring or enhancing θ wave function, which in turn strengthens the parietal modulation of the prefrontal cortex.

In summary, θ oscillations play a complex role in pain modulation by influencing pain perception, maintaining synchronization within the pain network, supporting neuroplasticity, and enhancing cognitive and emotional regulation functions. In patients with PSCP, abnormalities in θ wave function may significantly contribute to the persistence and intensification of pain. Therefore, exploring methods to restore θ wave function through tACS may offer novel directions and strategies for the effective treatment of PSCP.

### 5.2 Modulation of θ neural oscillations in sensorimotor cortex by tACS

The central nervous system of individuals experiencing chronic pain often demonstrates atypical neural oscillation patterns, with particular emphasis on the significance of θ wave oscillation abnormalities in contributing to central sensitization. Notably, alterations in θ oscillation states have been found to significantly correlate with pain alleviation achieved through deep brain stimulation (Peng and Tang, 2016; Luo et al., 2018). Consequently, we hypothesize that PSCP may be associated with the dysregulated synchronization of θ waves within the pain network. In exploring the modulatory effects of transcranial alternating current stimulation (tACS), the selection of the target area for modulation is a critical determinant, and the frequency of modulation is equally pivotal for the efficacy of tACS. Research indicates a propensity for electrical stimulation to enhance the causal coefficient within the θ frequency range post-stimulation. Specifically, α-tACS stimulation has been observed to reduce θ power in the Cg1 region (Mengfan, 2024). By restoring normal θ oscillations in the sensorimotor cortex, tACS holds the potential to suppress aberrant pain signaling and diminish pain sensitivity. Research has demonstrated that the offline effects of transcranial alternating current stimulation (tACS) are contingent upon alterations in neuroplasticity, specifically long-term potentiation (LTP) and long-term depression (LTD) (Vosskuhl et al., 2018), θ waves, which are intrinsically linked to neuroplasticity, can facilitate the reorganization of pain networks by inducing plasticity mechanisms such as LTP and LTD. tACS modulates the cerebral electric field via sinusoidal alternating currents to specifically target theta oscillations. This modulation can influence synaptic long-term plasticity and affect neuronal oscillations and synchronization, including inter-neuronal reconnection and functional reorganization, thereby enhancing neural network function. When the synchronization of these neural networks is restored to normative levels, the transmission and processing of pain signals may be optimized, effectively modulating brain function and consequently reducing pain perception (Maddison et al., 2023; Zhang et al., 2023). This modulation effectively regulates brain function and is crucial for the brain’s functional recovery post-stroke, with potential applications in pain management. Beyond the sensorimotor cortex, tACS can influence θ wave activity in other brain regions associated with pain and cognitive functions. Specifically, θ waves induced by tACS in the hippocampal regions can modulate memory functions, particularly in contexts of pain-induced cognitive burden or anxiety, and modulation of θ waves in the hippocampus may contribute to alleviating patients’ perceptions of pain (Chang et al., 2023; Hyman et al., 2003). Transcranial alternating current stimulation (tACS) influences θ wave activity in the prefrontal cortex, thereby impacting functions associated with emotion and decision-making (Booth et al., 2022). This modulation can alleviate pain-induced negative emotions and stress responses (Alekseichuk et al., 2016). The researchers demonstrated that the introduction of theta oscillations in the dorsolateral prefrontal cortex via transcranial alternating current stimulation (tACS) effectively modulated the propagation of transcranial magnetic stimulation (TMS)-induced neural activity in a phase-dependent manner. This finding further substantiates the causal role of neural oscillations in the modulation of neural signaling (Feher et al., 2017).

The sensorimotor cortex is a critical region for nociceptive processing, playing a role in the localization of pain, perception of its intensity, and discrimination of its properties. Neuromodulation of the primary motor cortex (M1) can influence neuraxial pathways at various levels to mitigate pain, while the excitability of the primary somatosensory cortex (S1) may be modulated through the M1 cortico-cortical pathway. Persistent plasticity in M1 and S1 can be facilitated by repetitive patterns of cortico-cortical fibers originating from events in the contralateral primary cortex (from S1 to M1 and M1 to S1) that are stimulus-evoked (Iriki et al., 1989; Enomoto et al., 2001; Frot et al., 2013). Both acute and tonic injurious stimuli, as well as chronic pain conditions, can alter motor cortical excitability (Duerden and Albanese, 2013; Burns et al., 2016). Abnormal cortical excitability induced by pain can be mitigated by reducing pain neuromodulation, which may be compromised following a stroke, resulting in altered pain perception. Within the sensorimotor cortex, θ waves are integral to the integration of sensory inputs and motor outputs, and they play a significant role in pain modulation. θ oscillations in the primary somatosensory cortex have been identified as predictive markers of pain in humans (Tan et al., 2021). Transcranial alternating current stimulation (tACS) can enhance synchronized neuronal activity by modulating θ oscillations in the sensorimotor cortex, thereby improving functions related to pain perception and motor control. Research indicates that patients with complex regional pain syndromes and neurogenic pain exhibit elevated baseline levels of delta and/or θ electroencephalogram (EEG) oscillations in the somatosensory cortex, which is related to pain localization, and in the orbital-frontotemporal cortex, which is linked to affective pain perception, when compared to healthy controls (Sarnthein and Jeanmonod, 2008; Walton et al., 2010). tACS has been shown to modulate neural activity by either enhancing or reducing the amplitude of θ oscillations and adjusting their phase, which in turn affects the synchronization of local neural networks. Additionally, tACS can synchronize neuronal activities, thereby improving signaling efficiency within specific brain regions, particularly within the pain modulation network. This synchronization effect may inhibit abnormal neural activities associated with chronic pain (Takeuchi, 2023).

### 5.3 Forms of stimulation for tACS

The form of tACS stimulation can be categorized into two forms: open-loop stimulation and closed-loop stimulation: in open-loop stimulation, the stimulation parameters of theta oscillations (e.g., frequency and intensity) are pre-set and are not adjusted based on the patient’s real-time neural activity during the treatment process, and are commonly used in preliminary clinical trials and basic research to test the effects of different stimulation parameters on pain relief. Open-loop stimulation has a more fixed effect and may be effective in some patients, but due to the lack of individualized adjustment, the effect may vary depending on the patient’s response. In contrast, closed-loop stimulation adjusts the stimulation parameters of theta oscillations based on real-time monitoring (e.g., EEG, pain scores, or neural activity). Stimulation intensity and frequency are dynamically adjusted according to the patient’s immediate response, allowing for personalized treatment. It is suitable for more complex pain management, especially when dealing with chronic, refractory pain. Closed-loop stimulation provides immediate feedback and adjustments as the patient’s neural status and pain response changes (Li Q. et al., 2023; Zrenner and Ziemann, 2024). Studies have shown that closed-loop stimulation significantly enhances treatment outcomes because it adjusts stimulation in real time to the patient’s neural activity, provides more precise neuromodulation, reduces side effects, and extends the duration of pain relief (Antony et al., 2022). Closed-loop stimulation showed better efficacy and lower individual differences for the treatment of post-stroke pain.

Due to the lack of real-time feedback and individualized modulation, the effect of open-loop stimulation may be more limited, and the efficacy may be less than expected, especially in complex neuropathic situations. However, it can still provide some degree of pain relief, especially in initial or milder patients. Closed-loop stimulation has better efficacy in the treatment of post-stroke pain (especially CPSP) due to its ability to dynamically adjust stimulation parameters to better match the patient’s neurologic status (Ting et al., 2021). Compared with open-loop stimulation, the closed-loop system is able to adjust the stimulation parameters according to the patient’s real-time neural activity, providing a personalized treatment plan and improving the therapeutic effect. And by monitoring neural activity in real time, the closed-loop system delivers stimulation only when needed, reducing unnecessary electrical stimulation and the risk of side effects. In addition, closed-loop stimulation can more effectively regulate abnormal neural activity, improving treatment efficiency and shortening treatment time (Sierra et al., 2023). By combining real-time monitoring techniques such as neuroimaging and EEG, closed-loop stimulation can provide more personalized, precise and effective treatment (Zelmann et al., 2020).

In summary, tACS can improve the synchronization of neural networks by modulating theta neural oscillations, involves multiple pain-related brain regions, modulates pain networks, and reduces central sensitization. tACS also differs in treatment between open-loop and closed-loop stimulation modes, with closed-loop stimulation showing superior therapeutic efficacy due to its ability to modulate and adapt to the patient’s neural responses in real time (Xu et al., 2024). Despite the limited number of clinical studies investigating the use of tACS for chronic pain relief, Bernardi et al. (2021) conducted a double-blind, randomized crossover study in which participants were randomly assigned to receive either tACS or transcranial random noise stimulation (tRNS) 5 days per week over a two-week period. In this study, the intervention group received tACS, while the control group was administered tRNS. The researchers established three measurement time points: T0 (baseline), T1 (post-stimulation), and T2 (1 month or 4 weeks post-stimulation). The findings indicated that tACS was associated with an increase in EEG α1 activity [(8–10) Hz] at T1 and a reduction in pain symptoms, as measured by a visual analog scale, at T1 compared to tRNS (Bernardi et al., 2021). Additionally, tACS stimulation was found to enhance the alpha oscillatory signal strength of electrodes located near the somatosensory area, and this enhancement was significantly correlated with a reduction in pain intensity (Ahn et al., 2019). Consequently, while tACS may hold potential for alleviating chronic pain, there remains insufficient evidence to support its efficacy in improving chronic pain following a stroke. tACS exhibits potential for modulating the pain network in the treatment of chronic post-stroke pain and demonstrates applicability across a broad spectrum of neurological and psychiatric disorders. However, further empirical studies are necessary to substantiate these findings and establish a scientific foundation for developing more precise and personalized tACS treatment protocols.

## 6 Discussion

A review of pertinent literature elucidates the significant role of θ oscillations in pain modulation and highlights the potential of tACS to modulate θ waves within the sensorimotor cortex to alleviate pain. This review not only synthesizes the principal findings and underlying mechanisms but also critically examines the limitations of existing studies and suggests directions for future research. Notably, θ waves are integral to pain perception and modulation, with their oscillatory activity being closely associated with the functional status of pain-related brain regions, such as the sensorimotor cortex, anterior cingulate gyrus, and insula. The θ oscillatory function of the sensorimotor cortex may be compromised in patients with PSCP, manifesting as reduced amplitude and abnormal network connections, which are closely associated with persistent pain perception. tACS through the phase coupling of external AC current, significantly enhances θ wave activity in the sensorimotor cortex and improves neural network synchronization. tACS not only effectively alleviates pain but also facilitates the recovery of neural function and plasticity by modulating θ oscillations. This modulation may be achieved through mechanisms such as synchronizing external and internal oscillations, promoting neural network reorganization, and inducing long-lasting effects.

### 6.1 Deficiencies and limitations

Although existing studies have made significant progress and demonstrate the considerable potential of tACS in pain modulation, they also reveal certain shortcomings and limitations in the research process, indicating that several challenges remain to be addressed. Currently, the majority of experimental and clinical studies on tACS for analgesia are characterized by small sample sizes, limited sample heterogeneity, and suboptimal statistical power, which calls into question the generalizability of their findings. Furthermore, there is a notable issue of insufficient parameter optimization. The optimization of tACS stimulation parameters, such as frequency, intensity, and duration, remains in its nascent stages, and variations in these parameters across different studies may compromise the comparability of their results. Additionally, the specific mechanisms underlying tACS’s effects have not been thoroughly investigated. While the modulation of θ oscillations by tACS has been preliminarily demonstrated, its precise neural mechanisms remain inadequately elucidated.

Given that neural synchronization effects are linked to endogenous neural oscillatory signals, the frequencies selected for analgesic studies are those associated with neural oscillations closely related to pain processing. Since the neural synchronization effect is related to the endogenous neural oscillatory signals, the stimulation frequency selected in the analgesic study is the frequency corresponding to the neural oscillatory signals that are closely related to pain processing. In the future, the modulation effect of tACS on pain at different frequencies (e.g., α-wave, *γ*-wave) can be further explored to study the specific effects of different frequencies on the neural network. Comparing the effects of tACS at different stimulation locations (e.g., prefrontal, parietal) to optimize the selection strategy of stimulation target areas (Tu et al., 2016; Vodovozov et al., 2018; Hu and Iannetti, 2019), Additionally, the combined effects of tACS with other non-invasive brain stimulation techniques, including transcranial direct current stimulation (tDCS) and transcranial magnetic stimulation (TMS), warrant further exploration. The potential synergistic mechanisms of tACS in conjunction with pharmacological interventions, psychotherapy (e.g., cognitive behavioral therapy, CBT), and physical therapy (e.g., visceral acupressure) should also be examined.

### 6.2 Potential application of tACS in PSCP

As a non-invasive and low-risk brain stimulation technique, tACS has become a hot research topic in the field of neuroscience in recent years due to its ability to precisely modulate neural activity in the brain. By applying a weak electric current, this technique modulates neural oscillation patterns in specific regions of the brain, especially theta wave activity in the sensorimotor cortex, and shows great potential for the treatment of neurological disorders. tACS provides an innovative therapeutic tool for a variety of neurological disorders by directly interfering with the relevant pathologic mechanisms. Compared with traditional pharmacologic or surgical treatments, tACS has significant advantages. While traditional treatments mostly rely on drugs or surgical means to temporarily relieve symptoms, tACS not only achieves short-term relief by inducing neuroplasticity in the brain, but also maintains its therapeutic effects in the long term by adjusting the structure of the neural network. Therefore, tACS is considered to have a broader application in chronic pain management, neurorehabilitation, and the treatment of other neurological disorders, especially in the areas of dealing with post-stroke chronic pain (PSCP), depression, anxiety, movement disorders, and cognitive disorders (Elyamany et al., 2021).

Even more important is the potential for personalized therapy with tACS. With the in-depth study of brain network characteristics and the emergence of advanced computational models, tACS will be able to design precise stimulation parameters based on the patient’s individualized neural network profile to maximize efficacy. This precise treatment approach is expected to overcome the limitations of traditional treatments and provide more efficient therapeutic results. Personalized neuromodulation also provides new ideas for the treatment of chronic and complex diseases. tACS has the potential to become a widely applicable “daily treatment,” especially in the treatment of neurological diseases that require long-term intervention, which can greatly improve the quality of life of patients, and provide more comprehensive and effective rehabilitation support for patients. Support. In addition, tACS is not limited to pain relief, but can also be used to improve cognitive function, emotional regulation, and enhance neuroplasticity. In recent years, studies have shown that tACS has positive effects in the treatment of psychological disorders such as depression and anxiety, and is able to improve emotional state and cognitive function by regulating neural activity (Grover et al., 2023). Compared with traditional treatments, the unique advantages of tACS in these areas are gradually being recognized in clinical applications.

By precisely modulating θ oscillations in the sensorimotor cortex, tACS provides a new direction for mechanism research and clinical treatment of neurological disorders such as PSCP. Although the current study is still advancing, preliminary results indicate that tACS not only shows significant efficacy in pain relief, but also demonstrates a wide range of potential applications in various aspects such as neuroplasticity and emotion regulation. This provides an important foundation for the further development of personalized treatment options in the future. With the strengthening of interdisciplinary cooperation and continuous technological innovation, tACS is expected to become an important tool in the field of neuromodulation. From precise individualized treatment to multi-modal integrated intervention, the wide application of tACS will greatly promote the change of medical treatment model, making the future treatment more efficient, personalized and integrated.

In the future, with the gradual deepening of the understanding of the complexity of brain networks and the mechanisms of interactions between brain regions, the combination of tACS with other brain modulation techniques will also open up new therapeutic horizons. For example, tDCS (transcranial direct current stimulation) and rTMS (repetitive transcranial magnetic stimulation) have achieved successful clinical applications to a certain extent, and the combination with tACS may bring even more significant therapeutic effects. The combination of tACS and other neuromodulation techniques can realize more delicate and comprehensive interventions through the linked regulation of different brain regions, further enhancing the therapeutic effects.

In conclusion, tACS, as an innovative neuromodulation tool, will provide new treatment options for patients with neurological disorders by virtue of its significant clinical efficacy, low risk and potential for personalized treatment. With more research, the application areas of tACS will be further expanded, and it will play an increasingly important role in multidisciplinary fields such as neuroscience, pain medicine, and psychotherapy.
